# Effects of risperidone on psychotic symptoms and cognitive functions in 22q11.2 deletion syndrome: Results from a clinical trial

**DOI:** 10.3389/fpsyt.2022.972420

**Published:** 2022-10-26

**Authors:** Caren Latrèche, Johanna Maeder, Valentina Mancini, Maude Schneider, Stephan Eliez

**Affiliations:** ^1^Developmental Imaging and Psychopathology Laboratory, Department of Psychiatry, University of Geneva School of Medicine, Geneva, Switzerland; ^2^Clinical Psychology Unit for Developmental and Intellectual Disabilities, Faculty of Psychology and Educational Sciences, University of Geneva, Geneva, Switzerland; ^3^Research Group Psychiatry, Department of Neuroscience, Center for Contextual Psychiatry, Katholieke Universiteit Leuven, Leuven, Belgium; ^4^Department of Genetic Medicine and Development, University of Geneva School of Medicine, Geneva, Switzerland

**Keywords:** 22q11 deletion syndrome, psychotic disorders, neuroprotective effect, cognition, antipsychotic treatment

## Abstract

**Background:**

Carriers of the 22q11.2 deletion syndrome (22q11DS) have an enhanced risk of developing psychotic disorders. Full-blown psychosis is typically diagnosed by late adolescence/adulthood. However, cognitive decline is already apparent as early as childhood. Recent findings in mice show that antipsychotic medication administered during adolescence has a long-lasting neuroprotective effect. These findings offer promising evidence for implementing preventive treatment in humans at risk for psychosis.

**Methods:**

We conducted a 12-week double-blind randomized controlled clinical trial with individuals with 22q11DS. Recruitment difficulties resulted in a final sample size of 13 participants (*n* = 6 treated with antipsychotics and *n* = 7 receiving placebo). We examined the response to treatment and assessed its short- and long-term effects on psychotic symptomatology using the Structured Interview for Psychosis-Risk Syndromes (SIPS) and cognitive measures.

**Results:**

First, two treated participants discontinued treatment after experiencing adverse events. Second, treated participants showed a short-term improvement in 33.3% of the SIPS items, mainly those targeting negative symptoms. Third, reliable improvements in at least one measure of working memory and attention were respectively found in 83.3 and 66.7% of treated participants.

**Conclusion:**

This is the first double-blind study to investigate the potential neuroprotective effect of antipsychotics in humans at risk for psychosis. Our preliminary results suggest that antipsychotic treatment may prevent long-term deterioration in clinical symptoms and cognitive skills. Yet, given the limited sample size, our findings need to be replicated in larger samples. To do so, future studies may rather adopt open-label or retrospective designs to ensure sufficient power.

**Clinical trial registration:**

[www.ClinicalTrials.gov], identifier [NCT04639960].

## Introduction

22q11.2 deletion syndrome (22q11DS) is a neurogenetic disorder which prevalence reaches as high as 1 in 2148 livebirths (1). The condition is associated with a 41% increased risk of developing schizophrenia by adulthood (2). The presentation of psychosis in 22q11DS is comparable to idiopathic psychosis, where its onset is understood as the last stage of an atypical neurodevelopment (3, 4). Studying 22q11DS therefore represents a unique opportunity to identify specific markers for the emergence of psychosis (5). One of the first apparent manifestations of psychosis lies in the cognitive development (6). In both deleted and non-deleted individuals, a global deterioration in reasoning skills [as measured by intellectual quotient (IQ) points] has been identified in early adolescence, prior the onset of psychotic symptoms (7–9). Moreover, such cognitive deficits are underpinned by several structural brain alterations which also precede psychosis onset affecting both cortical (10, 11) and subcortical structures (12, 13). These abnormalities are associated with developmental alterations in high-frequency oscillations, particularly in the gamma-band range [30–100 Hz (14, 15)], which synchronization allows for flexible communication between cortical regions (16).

In the last decades, a multitude of studies has allowed to better define the neuroanatomical and neuropsychological phenotypes in deleted individuals with psychosis. Yet currently, one major challenge is to develop early neuroprotective interventions to prevent the cerebral and cognitive deteriorations which predate psychosis (17). A recent study has highlighted the promising neuroprotective effect of selective serotonin reuptake inhibitors (SSRI) treatment in children and adolescents with 22q11DS (18). Participants treated with SSRIs showed improved IQ scores and increased cortical thickness in the frontal lobe and the hippocampus, which were more pronounced as treatment started early, suggesting the potential benefit to intervene during the critical developmental window of adolescence. Furthermore, a combination of SSRIs and atypical antipsychotics revealed stronger effects on cognitive and brain outcomes. However, the specific contribution of antipsychotics as a potential neuroprotective therapy is yet to be investigated in 22q11DS.

Recently, antipsychotic treatment has begun to attract attention as a promising neuroprotective intervention in 22q11DS. First evidence arose from one study in LgDel+/-mice, the homologous mice model of 22q11.2 deletion (19). The authors discovered deficits in parvalbumin interneurons (PVI) in LgDel+/-mice, which have a crucial role in the generation of gamma oscillations and in cognitive functioning (20). Interestingly, the severity of these deficits drastically depended on the developmental stage. While PVI hypofunction was restricted to the CA1 and subiculum regions of the hippocampus during early adolescence, it spread to medial prefrontal cortex (mPFC), basolatereal amygdala, and dorsal striatum from late adolescence onwards. By adulthood, these cerebral alterations became more pronounced and were associated with reduced power in the high-gamma range, along with impaired cognitive flexibility and episodic memory. To counteract this deterioration, the authors repeatedly administered a D2 receptor antagonist (risperidone) in ventral hippocampus or mPFC. Strikingly, their findings indicate a long-lasting rescue of PVI only during a specific time-window in late adolescence, which prevented cognitive and gamma-band deficits in LgDel+/- mice. As risperidone is a commonly used atypical antipsychotic, these results have important clinical implications for individuals with 22q11DS. When taken as a preventive treatment, antipsychotics may represent a promising intervention for counteracting the developmental alterations associated with the syndrome.

Antipsychotics are widely prescribed in deletion carriers to treat psychotic symptoms. The evidence for their effectiveness and safety in 22q11DS is yet limited. In a retrospective study (21), comparable rates of adverse events and improvement were showed between deleted and non-deleted individuals with psychosis. The authors concluded that antipsychotics are both effective and relatively safe in the management of psychotic symptoms. Nevertheless, their conclusion is limited by a small sample size, which is neither representative of the 22q11DS population nor comparable to the more substantial samples used in studies with non-deleted individuals. Moreover, as the adverse effects of antipsychotics are common manifestations observed in 22q11DS (e.g., weight gain, cardiac side effects; 22), no definite conclusions can be drawn regarding the safety and the tolerability of antipsychotics in 22q11DS.

In the present study, our aim was twofold. Firstly, to examine the response to atypical antipsychotic treatment (risperidone) in deletion carriers. Secondly, to investigate the short- and long-term effects of risperidone on psychotic symptoms and cognitive functions. Based on encouraging results on mice, we expected to find favorable effects of risperidone treatment on cognitive skills in deletion carriers. To test this hypothesis, we conducted a double-blind randomized controlled clinical trial (DBRCT) in deletion carriers. However, conducting a rigorous study with individuals affected by a relatively rare genetic condition proved to be tremendously challenging. Given the potential benefits for the families of individuals with 22q11DS, we have persevered in our efforts and carried out this study for 3 years and 7 months. Despite our strong commitment, we terminated this clinical trial, mainly due to challenges encountered in the recruitment process which resulted in a limited sample size. Nonetheless, given the paucity of pharmacological trials in 22q11DS, we present here novel and valuable evidence for the effects of risperidone on psychotic symptoms and cognitive functions in deletion carriers.

## Materials and methods

### Participants

**FIGURE 1:**
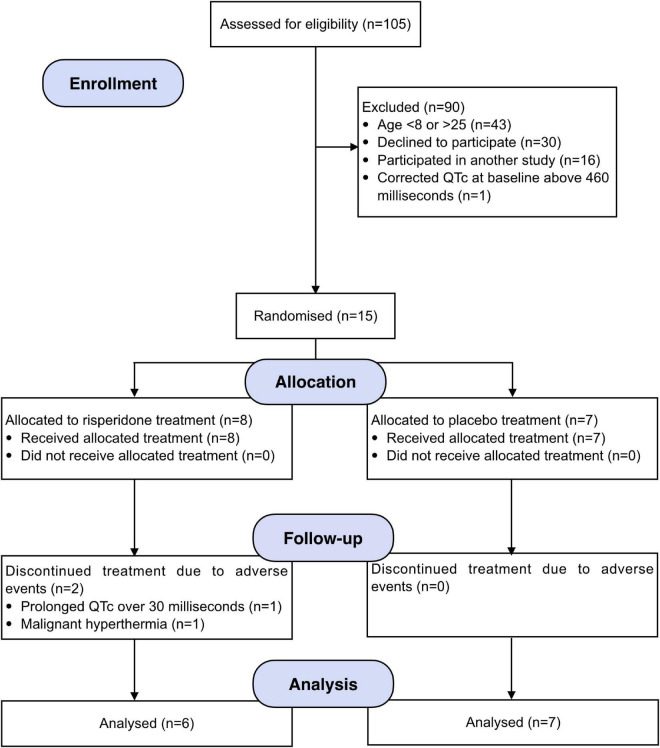
CONSORT flow diagram.

Participants were recruited from the longitudinal cohort of 22q11DS patients based in Geneva (see flow diagram in Figure 1). Sixteen participants (5 females) with 22q11DS were enrolled in this study and had a deletion confirmed by quantitative fluorescent polymerase chain reaction (QF-PCR). They were recruited from September 29*^th^* 2017 to May 1*^st^* 2021. Written informed consent based on protocols approved by the Swiss Ethical Committee of Geneva (CCER) and the Swiss Agency for Therapeutic Products (Swissmedic) was obtained from participants or parents (for participants younger than 18 years). Inclusion and exclusion criteria are available in Supplementary material.

Among the total sample, one participant was excluded due to long QTc (>460 ms) measured at baseline. Moreover, two participants discontinued the study. The first after experiencing a prolonged QTc interval (+103 ms) at Day 7. The second after experiencing malignant hyperthermia (body temperature increase of 1.3°C and trembling) at Day 25. For both cases, breaking the blind revealed active treatment, and complications resolved after discontinuation, without sequelae. The final sample included 6 participants in the risperidone group and 7 participants in the placebo group. Reasons for non-participation included lack of time to commit to the study, reluctance to take a preventive psychotropic treatment, and lack of evidence in humans with 22q11DS.

### Procedure

This study entailed three assessments (Supplementary Figure 2), conducted by a trained psychologist in Geneva. The first assessment (baseline) occurred 1 month before the treatment phase. A month later, treatment was introduced for a duration of 12 weeks. It took place under increased monitoring, with regular contacts organized between the examiner, the participant, and their caregivers. Frequent temperature checks and the completion of an electrocardiogram (ECG) at beginning of treatment were requested. The second and third visits occurred 7 days and 6 months after the end of treatment, and aimed to assess short- and long-term effects, respectively.

### Treatment

Participants were randomized to receive either placebo or risperidone. Treatment allocation was randomized by the pharmacy service of the Lausanne University Hospital (CHUV), such that investigators and participants were blind to the medication. Regarding dosage, three weight categories were defined (see Supplementary Table 1). Treatment was introduced and interrupted progressively. Unblinding procedure was performed after the third visit of the last enrolled participant.

### Materials

First, we investigated safety and tolerance to treatment by assessing reported side effects. Second, we evaluated the presence and severity of psychotic symptoms. Third, we examined the effects of treatment on several cognitive domains. The study protocol is comparable to that of one of our previous studies of Maeder et al. (22).

#### Safety and tolerance to treatment

Two ECGs were obtained: the first at baseline, and the second between Days 4 and 7 of treatment by the patient’s referring physician. We verified that no elongation of QTc (>30 ms) occurred after treatment onset.

A homemade questionnaire was sent to the participant and their caregivers on days of increasing or decreasing increments in dosage. We collected information regarding administration of treatment, temperature measurement and side effects. The evaluation of side effects was inspired by the Barkley Side Effect Rating Scale (23), with seven categories being investigated (cardiovascular, gastrointestinal, psychiatric, mood, neurologic, sleep disturbances, and other side effects). Severity of side effects was measured on a 5-intensity scale (1 = mild to 5 = severe).

#### Clinical assessment

We assessed the presence and the severity of psychotic symptoms at each visit using the Structured Interview for Psychosis-Risk Syndromes (SIPS), a clinical tool validated in the 22q11DS population (24, 25). The SIPS is comprised of symptoms belonging to four subscales (positive, negative, disorganization, and general), rated on a 7-severity scale (0 = absent to 6 = severe and psychotic).

#### Cognitive assessment

We administered several cognitive tasks at each visit targeting the following domains: planning, initiation, processing speed, updating, inhibition, cognitive flexibility, attention, and (verbal and nonverbal) learning and long-term memory. A description of each measure is available in Supplementary Table 2.

### Statistical analyses

Prior to the beginning of recruitment, a power analysis showed that a minimal sample size of *n* = 26 was needed to obtain 0.8 power, *p*-value of 0.05 and 0.5 mean effect size. Given our sample size of 13 participants, no statistical analysis could be performed to detect group differences. Considering our limited sample size and the within-group heterogeneity observed in cognitive measures (see Supplementary Figure 1), we opted for individual analyses. We assessed intra-individual change in clinical and cognitive measures after risperidone or placebo. We used the Reliable Change Index (RCI) to quantify the magnitude of short- and long-term change and determine whether it is clinically reliable or not (26). Using the Jacobson-Truax Method, we calculated the RCI formula for each variable, as follows:


$$R\,C\,I=\frac{X_{2}-X_{1}}{S\,E_{d\,i\,f\,f}}$$

where *X*_1_ represents the participant’s score at the first assessment (baseline), *X*_2_ represents the participant’s score at the second or third assessment (to assess short- or long-term change, respectively), and *SE*_*diff*_ is the standard error of difference between the two time-points. Reliable change is achieved if the index exceeds the threshold of ±1.96 (two-tailed 95% confidence interval). Beyond this threshold, the change is considered to not be only due to measurement error, and indicates either a reliable improvement or a reliable deterioration. Percentages were then computed based on measures that showed reliable change.

## Results

### Safety and tolerability of risperidone treatment in 22q11.2 deletion syndrome

Findings regarding safety and tolerability of treatment are shown on Supplementary Table 3 (see Supplementary Table 4 for the placebo sample). The ECGs completed during treatment revealed no significant increase (>30 ms) in QTc intervals. Temperature checks showed slight decreases and increases (<1°C).

**FIGURE 2:**
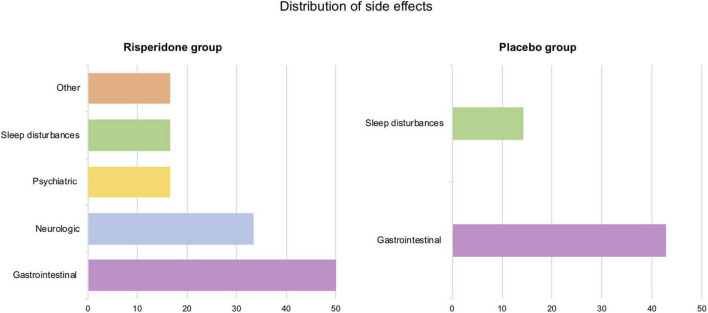
Distribution of side effects by category (in percentages) in the risperidone and the placebo groups.

The distribution of side effects in each group appears in Figure 2. Most participants from the treated group (66.67%) reported at least one side effect during treatment. The majority of side effects (40%) was reported when dosage increased. The most prevalent side effects affected the gastrointestinal and neurologic systems. Among treated participants, half experienced gastrointestinal disturbances, with stomachache of mild intensity being the most represented (66.67%). Two participants experienced neurologic side effects when dosage increased: one reported finger paresthesia and the other, headache. Regarding other categories, moderate sleep disturbances were reported once (16.67%) and mild hot flashes (with no associated fever) were observed twice by the same participant.

The placebo group also reported several side effects (42.86%). Gastrointestinal disturbances were the most represented (42.86%) as well, both at the beginning and the end of the treatment phase. We observed a slight prolongment in QTc in two participants, yet as no functional complaint was associated, treatment was continued in agreement with their respective referring physicians.

### Psychotic symptomatology

Results regarding the SIPS are in Supplementary Table 5 for the treated group and in Supplementary Table 6 for the placebo group. We first examined the severity scores of each subscale at baseline. The treated group tended to display greater mean severity in the positive subscale (*m* = 5.17) compared to the placebo group (*m* = 1.57), meaning that the potential for improvement in positive symptoms was lower in the placebo group. Comparable scores between groups were observed for the negative (*m* = 11 and *m* = 10.86, for the risperidone and placebo groups, respectively), disorganization (*m* = 4.17 and *m* = 4.43), and general subscales (*m* = 2.83 and *m* = 2.43).

#### Short-term effects

First, when considering the nineteen symptoms of the SIPS 1 week after treatment ended, a reliable improvement was found in 8.77% of the symptoms in treated participants (vs. 6.02% in non-treated participants). We observed a reliable deterioration in 1.75% of symptoms in the treated group (vs. 3.01% in the placebo group). Second, when only considering the four subscale scores, a reliable improvement of 33.3% was revealed in the treated group (vs. 14.29% in the placebo group). A reliable short-term deterioration was revealed in one treated participant (vs. none was found in the placebo group).

**FIGURE 3:**
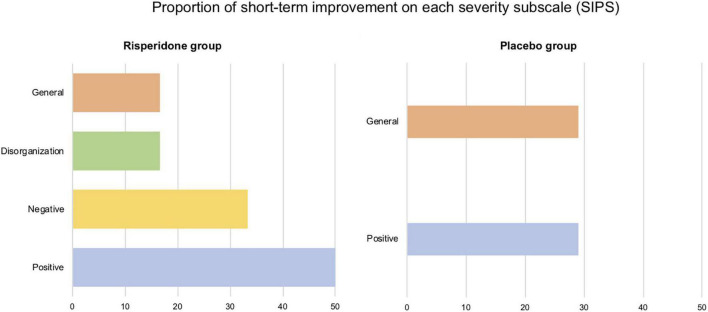
Proportion of short-term improvements on each severity subscale (positive, negative, disorganization, and general) of the Structured Interview for Psychosis-Risk Syndromes (SIPS). All (6/6) participants of the risperidone group displayed one improved score in at least one severity scale. In the placebo group, this proportion is 42.86% (3/7 participants).

All treated participants showed improved psychotic symptomatology in at least one of the four subscale scores, mostly in negative (50%) and positive (33.3%) symptoms. In the placebo group, three participants (42.86%) showed short-term improvements, in the positive and the general subscales, to the same extent (28.57%). The proportion of short-term improvements for both groups and on each subscale score appears in Figure 3.

#### Long-term effects

Six months after risperidone treatment, 9.65% of symptoms showed a reliable improvement (vs. 5.26% in the placebo group). A reliable deterioration was revealed in 0.88% of symptoms (vs. 9.02% in the untreated group). Regarding subscale scores, we observed an improvement of 12.5% in treated participants (vs. 3.57% in untreated participants). No long-term deterioration was observed in the treated group while a deterioration of 3.57% was observed in the placebo group.

More precisely, in the treated group, the proportion of long-term improvement was driven by Participant 6 only, who showed reliable decreases in severity of the negative, disorganization and general subscale scores. In the placebo group, improvements in subscale scores were also focused on one participant (Participant 2), who showed improvements in general symptomatology only.

### Cognitive measures

**FIGURE 4:**
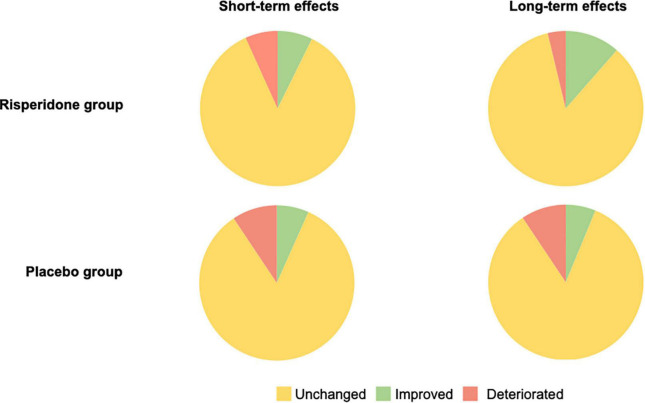
Short- and long-term effects treatment on all cognitive measures in the risperidone and placebo groups. Reliable change (i.e., improvements and deteriorations) is achieved if the Reliable Change Index (RCI) exceeds the threshold of ±1.96 (two-tailed 95% confidence interval).

Our findings regarding all cognitive measures are shown in Figure 4 (for further details see Supplementary Table 7 for the risperidone group and Supplementary Table 8 for the placebo group).

#### Short-term effects

We observed a short-term improvement in 7.29% of cognitive measures in the treated group, compared to 6.70% in the placebo group. Conversely, a reliable deterioration was noted in 6.77% of cognitive measures in the treated group (vs. 9.38% in the placebo group). In the treated group, most improved measures in the short-term follow-up occurred in two domains, namely working memory (WM; 50%) and attention (28.57%), both detailed below.

#### Long-term effects

Reliable long-term improvements were found in 11.46% of measures in the treated group (vs. 6.25% in the untreated group). We observed reliable deteriorations in 3.65% of cognitive measures in the treated group, compared to 9.38% in the untreated group. Most improvements in the treated group were again found in the domains of attention (40.91%) and WM (31.82%).

#### Working memory

Most treated participants (83.3%) showed a reliable improvement (both short- and long-term) in at least one WM measure. In the placebo group, short- and long-term improvements are, respectively observed in 14.29 and 42.86% of the sample.

Regarding short-term effects, 29.17% of WM measures were improved in the risperidone group (vs. 3.57% in the placebo group). For long-term effects, an identical proportion of improvement was found in WM measures in the treated group (vs. 10.71% in the placebo group). Yet, we observed a short-term reliable deterioration in 16.67% of WM measures whereas no short-term deterioration was noted in the placebo group. Reliable long-term deteriorations in the treated group affected 8.33% of WM measures, which all occurred in Participant 5. No reliable long-term deterioration was observed in the placebo group.

#### Attention

More than half (66.67%) of the treated group showed improvements in at least one attentional measure (vs. 42.87% in the placebo group). These proportions were the same in both short- and long-term comparisons. Note that Participant 6 (placebo group) started taking methylphenidate (MPH) just before the third visit. As MPH was found to have a favorable effect on attention and inhibition in 22q11DS (22), careful attention must be paid when examining long-term effects.

Regarding short-term effects, 7.41% of attentional measures were improved in the treated group (vs. 6.35% in the non-treated group). For long-term effects, the treated group showed an improvement in 16.67% of measures (vs. 11.11% in the placebo group). Reliable short-term deteriorations were observed in 7.41% of attentional measures in the treated group and were less prevalent in the placebo group (3.1%). Yet, in the risperidone and the placebo groups, we respectively, noted a decrease and an increase in long-term deteriorations (3.70 and 11.11%).

## Discussion

To our knowledge, this is the first study to investigate the effectiveness of risperidone in individuals with 22q11DS using a DBRCT design. We first aimed at examining the response to risperidone treatment. Second, we aimed at exploring short- and long-term effects of risperidone on psychotic symptoms and cognitive functions.

### Safety and tolerability to risperidone treatment

To assess response to risperidone in 22q11DS, we repeatedly documented changes in cardiac rhythm and body temperature, along with side effects over the course of treatment. In total, 2/8 participants enrolled in the treated group discontinued the study due to cardiac rhythm disorder and malignant hyperthermia, respectively listed as occasional (≥1/1000 to <1/100) and rare (≥1/10,000 to <1/1000) side effects of risperidone.^1^ Yet, a recent systematic review did not report these adverse events as being related to risperidone treatment in 22q11DS de Boer et al. (27). Conversely, the authors reported movement disorders, seizures, and weight gain as the main adverse effects of risperidone in 22q11DS, which were not observed in our sample. While this difference is likely due to our small sample size or/and to the short period of treatment, these findings suggest the wide array of adverse experiences associated with risperidone. Moreover, the high prevalence of mild to moderate side effects underline the importance of raising awareness on the great heterogeneity of risperidone response among deletion carriers. Although our limited sample size does not allow generalizations on the safety of antipsychotics in 22q11DS, our results support the implementation of an increased treatment monitoring, especially the use of ECG to monitor QTc interval. Indeed, one of the main risk factors of QTc prolongation include cardiovascular anomalies (28, 29) which reach a prevalence as high as 75% in the 22q11DS population (30, 31).

### Effects on psychotic symptomatology

The presence and severity of psychotic symptoms was assessed with the SIPS at each visit. We found greater short-term improvements in subscale scores in the treated group compared to the placebo group, mainly in negative symptoms (particularly avolition). This finding was unexpected, as antipsychotics are reported to have limited effects on primary negative symptoms (32), which are especially observed in 22q11DS. Indeed, Schneider et al. (33) found elevated rates of negative symptoms (73.9%) which severity predicted psychosis-risk and correlated with low global functioning. Regarding long-term effects, we observed that most improvements in subscale scores were not maintained 6 months after treatment, except for Participant 6 who showed increased improvements over time. Although these effects seem mostly transient, future studies will have to further investigate the effects of preventive antipsychotic treatment on negative symptoms in 22q11DS given the potential clinical implications.

Regarding short-term deteriorations, lower rates were found in the treated group compared to the placebo group when considering all symptoms. Interestingly, the rates of deterioration tended to decrease over time in the treated group, while they tended to increase in the placebo group. The lack of worsening symptoms over time in treated participants is especially striking as they experienced some attenuated symptoms at baseline. Indeed, this finding goes against the neurodevelopmental trajectory of schizophrenia, characterized by a progression from prodromal state to psychosis (3). Therefore, while this result needs to be replicated with larger sample size, it suggests that early antipsychotic intervention may reduce or prevent clinical deterioration.

### Effects on cognitive functions

We assessed the effects of risperidone on cognitive functions using several measures. Both groups showed comparable improvements a week after interrupting treatment. Yet, 6 months later, beneficial effects seemed to increase in the treated group while they seemed stable in the placebo group. While a learning effect may be present for some short-term results given the proximity of assessments, it is likely that this effect was reduced for long-term results. Interestingly, these findings mirror those on psychotic symptomatology, indicating a decreasing number of cognitive and clinical deteriorations over time in the treated group.

Moreover, WM and attention were the domains with the most improvements in treated participants. Strikingly, deficits in both domains characterize the cognitive profile of both 22q11DS (6, 34) and idiopathic schizophrenia (3, 35). Our findings are supported by previous studies in non-deleted individuals with psychosis which investigated the effects of antipsychotics on both verbal and spatial components of WM. One previous study (36) found that administering risperidone to chronic patients with schizophrenia led to reliable improvements in verbal WM performance after 4 and 8 weeks of treatment compared to patients administered other atypical antipsychotics. In another study (37), the authors conducted a DBRCT to compare the effects of risperidone and clozapine on spatial WM in patients with moderate treatment-resistant schizophrenia. After 17 and 29 weeks of treatment, reliable improvements in spatial WM performance were found in patients treated with risperidone, whereas with clozapine, performance decreased over time. The authors hypothesized that risperidone induced a beneficial effect on WM through its increased dopamine activity in the PFC, whereas antipsychotics with a strong anticholinergic effect like clozapine are associated with cognitive deficits in patients with schizophrenia (38). Previous studies indicate favorable effects of risperidone on WM in patients with chronic or treatment-resistant schizophrenia. Our findings yet seem to indicate that such improvements may already be observed in earlier stages of psychosis, i.e., in at-risk patients. In addition, the same rate of improvements was found at long-term follow-up, suggesting the potential prolonged effects of treatment.

Compared to WM, short-term improvements in attentional skills were less marked in the treated group. This is in line with a previous review reporting less consistent effects of risperidone on attention compared to WM (39). After 6 months, we found greater improvements and less deteriorations in attentional measures compared to the non-treated group. However, based on the scores from the Conners’ Continuous Performance Test 3^rd^ edition [(40); Supplementary Table 2], we could not distinguish any specific effects on attention measures (e.g., on inattention, impulsivity, vigilance, or sustained attention). Yet, our preliminary results may pave the way for further research on the effects of risperidone on attention in 22q11DS, given the increased prevalence and the persistence of ADHD in this population (2, 41, 42).

### Limitations

The main limitation of this study is our small sample size, which prevented statistical group comparisons. While DBRCTs are recognized as the gold-standard for studying treatment effectiveness, they appear as unsuitable in a rare condition like 22q11DS. Future studies may adopt less sophisticated designs (e.g., retrospective studies) to enable sufficient power and the formulation of practical therapeutic guidelines.

Another important limitation is that we could not control for the effect of concomitant psychotropic medication, taken by 5/13 participants (2/6 and 3/7 in the treated and placebo groups, respectively). As SSRIs and MPH significantly improve cognitive functions in 22q11DS (18, 22), concurrent treatment likely had an effect on our findings. The presence of multiple treatments that could interfere with the outcome measures stresses the need for larger datasets and alternative study designs.

## Conclusion

Our study offers encouraging evidence on the neuroprotective effects of preventive risperidone treatment, with deletion carriers showing less long-term deteriorations in cognitive functions and psychotic symptoms. Improvements were targeted at negative symptoms, WM, and attention, all identified as being altered in 22q11DS. Therefore, our study adds to emerging evidence that early pharmacological interventions may prevent the typical deteriorations in patients at-risk for psychosis.

## Data availability statement

The datasets presented in this article are not readily available because they contain information that could compromise the privacy of research participants. Requests to access the datasets should be directed to corresponding author.

## Ethics statement

The studies involving human participants were reviewed and approved by the Swiss Ethical Committee of Geneva (CCER). Written informed consent to participate in this study was provided by the participants’ legal guardian/next of kin. Written informed consent was obtained from the individual(s), and minor(s)’ legal guardian/next of kin, for the publication of any potentially identifiable images or data included in this article.

## Author contributions

CL, JM, VM, MS, and SE: investigation, writing – review, and editing. CL, JM, VM, and SE: data acquisition. CL: formal analysis and writing – original draft. All authors contributed to the article and approved the submitted version.

## Footnotes

1
https://compendium.ch
