# Supplementary material for: Effects of risperidone on psychotic symptoms and cognitive functions in 22q11.2 deletion syndrome: Results from a clinical trial
Source: Front Psychiatry. 2022 Oct 26;13:972420. doi: 10.3389/fpsyt.2022.972420 (PMC9643534; doi:10.3389/fpsyt.2022.972420)
Supplement: Supplementary file 1 [file Data_Sheet_1.docx]

Supplementary material

- Inclusion and exclusion criteria

The inclusion criteria for this study were as follows:

1. Male or female with confirmed 22q11DS diagnosis
2. Minimum age of 11 years or maximum age of 25 years and 11 months.
3. Sufficient verbal expression and comprehension skills to understand and follow instructions based on initial interview.

The exclusion criteria were:

1. Hypersensitivity to active substance.
2. Lifetime history of psychotic symptoms requiring antipsychotic treatment for over one month.
3. Corrected QT (QTc) distance at baseline electrocardiogram above 460ms or prolongment of QTc during treatment phase (Days 4-7) superior to 30ms, associated with functional complaint, which represent an increased risk for sudden heart failure.
4. Pregnancy or breastfeeding.

- Statistical analyses

Using the Jacobson-Truax Method, we calculated the RCI formula for each variable, as follows:

$RCI =$ $\frac{X_{2} - X_{1}}{{SE}_{diff}}$

where $X_{1}$ represents the participant’s score at the first assessment (baseline),$X_{2}$ represents the participant’s score at the second or third assessment (to assess short- or long-term change, respectively), and ${SE}_{diff}$ is the standard error of difference between the two time-points. Reliable change is achieved if the index exceeds the threshold of ±1.96 (two-tailed 95% confidence interval). Beyond this threshold, the change is considered to not be only due to measurement error, and indicates either a reliable improvement or a reliable deterioration. Percentages were then computed based on measures that showed reliable change.

- Supplementary Tables (S1-S8)

Supplementary Table S1. Risperidone treatment schedule per weight categories.

| Weight  category | Week 1  Progressive introduction | | | | Weeks 2 to 10  Maximal dosage of treatment | | | | Weeks 11 and 12  Progressive interruption | | | |
| --- | --- | --- | --- | --- | --- | --- | --- | --- | --- | --- | --- | --- |
|  | 0.25 mg | 0.50  mg | 0.75  mg | 1  mg | 0.25 mg | 0.50 mg | 0.75 mg | 1  mg | 0.25 mg | 0.50 mg | 0.75 mg | 1  mg |
| <50 kg | 7 days | - | - | - | - | 63 days | - | - | 14 days | - | - | - |
| 51-70 kg | 3 days | 4  days | - | - | - | - | 63 days | - | 7 days | 7 days | - | - |
| >70 kg | 3 days | 4  days | - | - | - | - | - | 63 days | 7 days | 7 days | - | - |

| **Cognitive domain** | **Cognitive task** | **Variable** |
| --- | --- | --- |
| Planning | Tower of London | Total correct scores |
| Initiation | Verbal Fluency | Number of animals produced |
|  | Figural Fluency | Number of different designs produced |
| Processing speed | Coding | Number of codes produced |
|  | Symbol Search | Number of symbols identified |
| Updating | Digit span | Forward span |
|  |  | Backward span |
|  | Letter-Number sequencing | Letter-number span |
|  | Spatial Working Memory^1^ | Between Errors (8 boxes) |
| Inhibition | Stroop | Inhibition ratio |
|  | Stop-Signal Task^1^ | Stop-Signal RT |
| Flexibility | Color Trails Test | Flexibility ratio |
|  | Intra-Extra Dimensional^1^ | Extra-Dimensional shift errors |
| Attention | CPT-3 | Omissions |
|  |  | Commissions |
|  |  | HRT SD |
|  |  | Perseverations |
|  |  | Detectability |
|  |  | Variability |
|  |  | HRT |
|  |  | HRT Block Change |
|  |  | HRT ISI Change |
| Learning and long-term memory | Modified 15 signs and 15 words (visual modality) | Learning rate |
|  |  | Retention 30 min |
|  |  | Retention 1 day |
|  |  | Retention 1 week |
|  |  | Retention 1 month |
|  | Modified 15 signs and 15 words (verbal modality) | Learning rate |
|  |  | Retention 30 min |
|  |  | Retention 1 day |
|  |  | Retention 1 week |
|  |  | Retention 1 month |

Supplementary Table S2. Description of cognitive measures. Detailed description and interpretation are available in Maeder et al. (2021b).

^1^Computerized measures of cognition using Cambridge Neuropsychological Test Automated Battery (CANTAB) tasks.

Supplementary Table S3. Characteristics of participants in the risperidone group.

| **Case** | **Age at**  **baseline** | **Sex** | **Comorbid**  **psychotropic treatment** | **Weight category** | **QTc (Δ)^1^** | **Temperature check (Δ)^2^** | **Side effect category^3^** | | | | | | |
| --- | --- | --- | --- | --- | --- | --- | --- | --- | --- | --- | --- | --- | --- |
|  |  |  |  |  |  |  | **Cardiovascular** | **Gastrointestinal** | **Psychiatric** | **Mood** | **Neurologic** | **Sleep disturbances** | **Other** |
| 1 | 16 | M | - | 51-70kg | +6ms | + 0°C | - | - | - | - | - | - | - |
| 2 | 22 | M | - | 51-70kg | +3ms | + 0.7°C | - | - | - | - | - | - | - |
| 3 | 14 | M | Psychostimulant | < 50kg | +6ms | -0.2°C | - | Stomach ache  (I=1, D8) | - | - | Paresthesia  (I=1, D8) | - | Hot flashes  (I=1, D8+D85) |
| 4 | 14 | F | - | < 50kg | +1ms | NA | - | Appetite increase  (I=3, D71) | Anxiety, irritability  (I=3, D71) | - | - | - | - |
| 5 | 12 | M | - | < 50kg | +7ms | - 0.1°C | - | Stomach ache  (I=1, D78) | - | - | Headache  (I=2, D8)  (I=1, D78) | - | - |
| 6 | 24 | F | Antiepileptic | 51-70kg | NA | - 0.5°C | - | - | - | - | - | Difficulties in falling asleep (I=3, D1) | - |
|  |  |  | Antidepressant |  |  |  |  |  |  |  |  |  |  |

^1^QTc (Δ): difference in milliseconds in the corrected QT interval between baseline measure and measure during treatment (Day 4^th^ to 7^th^ of treatment). ^2^Temperature expressed in Celsius degrees. ^3^ I= side effect intensity (scaled from 1 to 5) observed at D= day of treatment. NA = missing data. ^4^Psychostimulant treatment started before the third visit assessing long-term effects.

Supplementary Table S4. Characteristics of participants in the placebo group.

| **Case** | **Age at**  **baseline** | **Sex** | **Comorbid**  **psychotropic treatment** | **QTc (Δ)^1^** | **Temperature check (Δ)^2^** | **Side effect category^3^** | | | | | | |
| --- | --- | --- | --- | --- | --- | --- | --- | --- | --- | --- | --- | --- |
|  |  |  |  |  |  | **Cardiovascular** | **Gastrointestinal** | **Psychiatric** | **Mood** | **Neurologic** | **Sleep disturbances** | **Other** |
| 1 | 23 | M | - | -37ms | NA | - | Appetite increase  (I=1, D4) | - | - | - | - | - |
| 2 | 23 | M | - | +35ms | +0°C | - | - | - | - | - | - | - |
| 3 | 20 | M | - | +32ms | +0.4°C | - | - | - | - | - | - | - |
| 4 | 20 | M | Psychostimulant | NA | +0.2°C | - | Constipation  (I=3, D78) | - | - | - | Tiredness  (I=1, D4) | - |
|  |  |  | Antidepressant |  |  |  |  |  |  |  |  |  |
| 5 | 14 | M | Psychostimulant | -1ms | -1.2°C | - | Appetite increase  (I=1, D4)  (I=2, D78) | - | - | - | - | - |
|  |  |  | Antidepressant |  |  |  |  |  |  |  |  |  |
|  |  |  | Anxiolytic |  |  |  |  |  |  |  |  |  |
| 6 | 12 | M | Psychostimulant^4^ | -6ms | -0.1°C | - | - | - | - | - | - | - |
| 7 | 15 | F | - | +13ms | -0.1°C | - | - | - | - | - | - | - |

Supplementary Table S5. Presence and severity of psychotic symptoms (SIPS) at baseline and magnitude of change in the risperidone group. Reliable improvement (I) or deterioration (D) is indicated if Reliable Change Index (RCI) > ± 1.96.

|  | **Risperidone group** | | | | | | | | | | | | | | | | | |  |
| --- | --- | --- | --- | --- | --- | --- | --- | --- | --- | --- | --- | --- | --- | --- | --- | --- | --- | --- | --- |
| **Severity scales** | **Symptoms^1^** | **Participant 1** | | | **Participant 2** | | | **Participant 3** | | | **Participant 4** | | | **Participant 5** | | | **Participant 6** | | |
|  |  | **Baseline^2^** | **ST** | **LT** | **Baseline** | **ST** | **LT** | **Baseline** | **ST** | **LT** | **Baseline** | **ST** | **LT** | **Baseline** | **ST** | **LT** | **Baseline** | **ST** | **LT** |
| Positive | Total | 2 | **-2.29* (I)** | -1.75 | 1 | 0 | 0 | 7 | 0 | -0.87 | 6 | 0 | 0 | 11 | **-2.29* (I)** | 1.75 | 4 | -1.14 | -0.87 |
|  | P1 | 0 | 0 | 0 | 1 | 0 | 0 | 1 | 0 | 0 | 2 | 0 | 0 | 3 | 0 | **3.41* (D)** | 2 | **-3.90* (I)** | **-3.41* (I)** |
|  | P2 | 0 | 0 | 0 | 0 | 0 | 0 | 2 | 0 | 0 | 2 | 0 | 0 | 4 | **-3.39* (I)** | 0 | 2 | 0 | 0 |
|  | P3 | 0 | 0 | 0 | 0 | 0 | 0 | 1 | 0 | NA | 0 | 0 | 0 | 0 | 0 | 0 | 0 | 0 | 0 |
|  | P4 | 2 | **-3.80*(I)** | **-3.80 * (I)** | 0 | 0 | 0 | 3 | 0 | 0 | 2 | 0 | 0 | 4 | 0 | 0 | 0 | 0 | 0 |
|  | P5 | 0 | 0 | 0 | 0 | 0 | 0 | 0 | 0 | 0 | 0 | NA | NA | 0 | 0 | 0 | 0 | 0 | 0 |
| Negative | Total | 10 | **-2.24* (I)** | -0.30 | 6 | **-2.24* (I)** | -0.30 | 9 | **-2.24* (I)** | -0.30 | 12 | **4.48* (D)** | 0.60 | 14 | 0 | 0.30 | 15 | 0 | **-2.69* (I)** |
|  | N1 | 1 | 0 | 0 | 1 | 0 | 0 | 2 | 0 | 0 | 2 | 0 | 0 | 2 | 0 | 0 | 4 | 0 | **-7.43* (I)** |
|  | N2 | 1 | **-2.62*(I)** | -0.94 | 2 | **-2.62* (I)** | -0.94 | 1 | **-2.62*(I)** | -0.94 | 2 | **2.62* (D)** | 0.94 | 3 | 0 | 0.94 | 3 | 0 | **-2.81* (I)** |
|  | N3 | 2 | 0 | 0 | 1 | 0 | 0 | 1 | 0 | 0 | 2 | 0 | 0 | 3 | 0 | 0 | 2 | 0 | **-2.99* (I)** |
|  | N4 | 0 | 0 | 0 | 0 | 0 | 0 | 0 | 0 | 0 | 1 | 0 | 0 | 1 | 0 | 0 | 0 | 0 | 0 |
|  | N5 | 4 | 0 | 0 | 1 | 0 | 0 | 3 | 0 | 0 | 4 | 0 | 0 | 4 | 0 | 0 | 4 | 0 | -1.90 |
|  | N6 | 2 | 0 | 0 | 1 | 0 | 0 | 2 | 0 | 0 | 1 | **3.21* (D)** | 1.15 | 1 | 0 | 0 | 2 | 0 | **-2.30* (I)** |
| Disorganization | Total | 3 | 0 | 0 | 3 | **-2.90* (I)** | -1.76 | 5 | 0 | 0 | 4 | **-2.90* (I)** | -1.76 | 5 | 0 | 0 | 5 | **-2.90* (I)** | **-3.52* (I)** |
|  | D1 | 0 | 0 | 0 | 0 | 0 | 0 | 1 | 0 | 0 | 0 | 0 | 0 | 0 | 0 | 0 | 0 | 0 | 0 |
|  | D2 | 0 | 0 | 0 | 0 | 0 | 0 | 0 | 0 | 0 | 0 | 0 | 0 | 0 | 0 | 0 | 0 | 0 | 0 |
|  | D3 | 2 | 0 | 0 | 2 | **-3.13* (I)** | **-3.13* (I)** | 3 | 0 | 0 | 2 | 0 | 0 | 4 | 0 | 0 | 4 | **-3.13* (I)** | **-3.13* (I)** |
|  | D4 | 1 | 0 | 0 | 1 | 0 | 0 | 1 | 0 | 0 | 2 | NA | NA | 1 | 0 | 0 | 1 | **-2.12* (I)** | **-2.12* (I)** |
| General | Total | 2 | -1.26 | -0.68 | 0 | 1.26 | 0 | 2 | 1.26 | -0.68 | 1 | 0 | 0.68 | 4 | 0 | 0.68 | 8 | **-5.06* (I)** | **-4.07* (I)** |
|  | G1 | 2 | -1.76 | **-3.16* (I)** | 0 | 0 | 0 | 1 | 0.88 | -1.58 | 0 | 0 | 0 | 2 | 0 | 0 | 2 | 0 | 0 |
|  | G2 | 0 | 0 | 0 | 0 | 0 | 0 | 0 | 0 | 0 | 0 | 0 | 0.86 | 1 | 0 | 0.86 | 3 | **-16.21*(I)** | **-2.59* (I)** |
|  | G3 | 0 | 0 | 0 | 0 | 0 | 0 | 0 | 0 | 0 | 0 | 0 | 0 | 0 | 0 | 0 | 0 | 0 | 0 |
|  | G4 | 0 | 1.79 | 0.78 | 0 | 1.79 | 0 | 1 | 0 | 0 | 1 | 0 | 0 | 1 | 0 | 0 | 3 | 0 | **-2.35* (I)** |

^1^Symptoms: P1= Unusual Thought Content/Delusional Ideas, P2= Suspiciousness/Persecutory Ideas, P3= Grandiosity, P4= Perceptual Abnormalities/ Hallucinations, P5= Disorganized Communication, N1= Social Anhedonia, N2= Avolition, N3= Expression of Emotion, N4= Experience of Emotions and Self, N5= Ideational Richness, N6= Occupational Functioning, D1= Odd Behavior or Appearance, D2= Bizarre Thinking, D3= Trouble with Focus and Attention, D4= Personal Hygiene, G1= Sleep Disturbance, G2= Dysphoric Mood, G3= Motor Disturbances, G4= Impaired Tolerance to Normal Stress. ^2^Baseline scores are rated on a 7-severity scale (from 0=absent to 6=severe and psychotic).

Supplementary Table S6. Presence and severity of psychotic symptoms (SIPS) at baseline and magnitude of change in the placebo group. Reliable improvement (I) or deterioration (D) is indicated if Reliable Change Index (RCI) > ± 1.96. NA = insufficient variance to compute RCI.

|  | **Placebo group** | | | | | | | | | | | | | | | | | | | | |  |
| --- | --- | --- | --- | --- | --- | --- | --- | --- | --- | --- | --- | --- | --- | --- | --- | --- | --- | --- | --- | --- | --- | --- |
| **Severity scales** | **Symptoms^1^** | **Participant 1** | | | **Participant 2** | | | **Participant 3** | | | **Participant 4** | | | **Participant 5** | | | **Participant 6** | | | **Participant 7** | | |
|  |  | **Baseline^2^** | **ST** | **LT** | **Baseline** | **ST** | **LT** | **Baseline** | **ST** | **LT** | **Baseline** | **ST** | **LT** | **Baseline** | **ST** | **LT** | **Baseline** | **ST** | **LT** | **Baseline** | **ST** | **LT** |
| Positive | Total | 0 | 0 | 0 | 0 | 0 | 1.50 | 2 | -0.78 | -1.50 | 0 | 0 | 0 | 5 | **-3.57* (I)** | 1.50 | 3 | **-3.57* (I)** | -1.50 | 1 | 1.78 | 0 |
|  | P1 | 0 | 0 | 0 | 0 | 0 | 0 | 0 | 0 | 0 | 0 | 0 | 0 | 2 | 0 | 0 | 1 | **-2.50* (I)** | 0 | 0 | 0 | 0 |
|  | P2 | 0 | 0 | 0 | 0 | 0 | **3.50* (D)** | 1 | 0 | 0 | 0 | 0 | 0 | 1 | 0 | 0 | 1 | 0 | 0 | 1 | **6.00* (D)** | 0 |
|  | P3 | 0 | 0 | 0 | 0 | 0 | 0 | 0 | 0 | 0 | 0 | 0 | 0 | 0 | 0 | NA | 0 | 0 | 0 | 0 | 0 | 0 |
|  | P4 | 0 | 0 | 0 | 0 | 0 | 0 | 1 | NA | **-2.11* (I)** | 0 | 0 | 0 | 2 | NA | 0 | 1 | NA | **-2.11* (I)** | 0 | 0 | 0 |
|  | P5 | 0 | 0 | 0 | 0 | 0 | 0 | 0 | 0 | 0 | 0 | 0 | 0 | 0 | 0 | 0 | 0 | 0 | 0 | 0 | 0 | 0 |
| Negative | Total | 19 | 1.18 | -0.45 | 6 | 0 | 1.36 | 10 | -0.59 | 0 | 10 | -1.77 | -1.36 | 10 | 0.59 | 1.82 | 11 | 0.59 | 1.36 | 10 | 1.18 | -0.45 |
|  | N1 | 5 | 0 | 0 | 2 | 0 | 0 | 1 | 0 | 0.89 | 2 | -1.05 | -0.89 | 1 | 1.05 | 1.79 | 2 | 0 | 0.89 | 2 | 0 | 0 |
|  | N2 | 1 | **3.82* (D)** | **4.59* (D)** | 0 | 0 | 0 | 1 | 0 | 0 | 1 | 0 | 0 | 2 | 0 | **2.29* (D)** | 1 | 1.91 | **2.29* (D)** | 1 | 0 | 0 |
|  | N3 | 4 | 0 | 0 | 2 | 0 | 1.56 | 2 | 0 | 0 | 3 | **-2.50* (I)** | -1.56 | 2 | 0 | 0 | 2 | 0 | 0 | 2 | 0 | 0 |
|  | N4 | 2 | 0 | 0 | 0 | 0 | 0 | 0 | -1.94 | 0 | 0 | 0 | 0 | 0 | 0 | 0 | 0 | 0 | 0 | 0 | **2.50* (D)** | **2.50* (D)** |
|  | N5 | 4 | 0 | 0 | 2 | 0 | **5.32* (D)** | 4 | 0 | 0 | 1 | **-9.56* (I)** | **-5.32* (I)** | 4 | 0 | 0 | 4 | 0 | 0 | 4 | 0 | 0 |
|  | N6 | 3 | 0 | **-2.29* (I)** | 0 | 0 | 0 | 2 | 0 | -0.76 | 3 | 0 | 0 | 1 | 0 | 0.76 | 2 | 0 | 0.76 | 1 | 1.94 | 0 |
| Disorganization | Total | 9 | 0 | -0.49 | 4 | -0.55 | -0.49 | 3 | 0 | -0.45 | 4 | 0 | 0 | 4 | 0 | 0.49 | 3 | 0.55 | 1.47 | 4 | -0.55 | -0.49 |
|  | D1 | 2 | 0 | 0 | 0 | 0 | 0 | 0 | 0 | 0 | 0 | 0 | 0 | 0 | 0 | **2.11* (D)** | 0 | 0 | **2.11* (D)** | 0 | 0 | 0 |
|  | D2 | 1 | 0 | 0 | 0 | 0 | 0 | 0 | 0 | 0 | 0 | 0 | 0 | 0 | 0 | 0 | 0 | 0 | 0 | 0 | 0 | 0 |
|  | D3 | 3 | 0 | **-3.50* (I)** | 3 | **-3.50* (I)** | 0 | 2 | 0 | 0 | 3 | 0 | 0 | 3 | 0 | 0 | 2 | 0 | 0 | 2 | 0 | 0 |
|  | D4 | 3 | 0 | 0 | 1 | 0 | -1.16 | 1 | 0 | 0 | 1 | 0 | 0 | 1 | 0 | 0 | 1 | 1.56 | **2.31* (D)** | 2 | -1.56 | -1.16 |
| General | Total | 4 | **-1.97* (I)** | 0 | 2 | -1.11 | **-1.97* (I)** | 0 | 0 | 0 | 1 | 0 | 0 | 6 | -1.11 | -0.98 | 2 | **-2.23* (I)** | -0.98 | 2 | 0 | **1.97* (D)** |
|  | G1 | 3 | -1.76 | 0 | 2 | **-2.08* (I)** | **-3.37* (I)** | 0 | 0 | 0 | 0 | 0 | 0 | 3 | **-2.08* (I)** | -1.69 | 2 | **-4.16* (I)** | **-3.37* (I)** | 1 | **-2.08* (I)** | -1.69 |
|  | G2 | 0 | 0 | 0 | 0 | 0 | 0 | 0 | 0 | 0 | 0 | 0 | 0 | 1 | 0 | 0 | 0 | 0 | 1.73 | 0 | **3.52* (D)** | **3.47* (D)** |
|  | G3 | 0 | 0 | 0 | 0 | 0 | 0 | 0 | 0 | 0 | 0 | 0 | 0 | 0 | 0 | 0 | 0 | 0 | 0 | 0 | 0 | 0 |
|  | G4 | 1 | 0 | 0 | 0 | 0 | 0 | 0 | 0 | 0 | 1 | 0 | 0 | 2 | 0 | **2.11* (D)** | 0 | 0 | **2.11* (D)** | 1 | 0 | 0 |

^1^Baseline scores are rated on a 7-severity scale (from 0=absent to 6=severe and psychotic).

Supplementary Table S7. Magnitude of change in cognitive performance in participants of the risperidone group. Reliable improvement (I) or deterioration (D) is indicated in bold if Reliable Change Index (RCI) > ± 1.96. NA= missing data.

|  | | | **Risperidone group** | | | | | | | | | | | |
| --- | --- | --- | --- | --- | --- | --- | --- | --- | --- | --- | --- | --- | --- | --- |
| **Cognitive domain** | **Cognitive task** | **Variable** | **Participant 1** | | **Participant 2** | | **Participant 3** | | **Participant 4** | | **Participant 5** | | **Participant 6** | |
|  |  |  | **ST** | **LT** | **ST** | **LT** | **ST** | **LT** | **ST** | **LT** | **ST** | **LT** | **ST** | **LT** |
| Planning | Tower of London | Total moves | 1.55 | **2.37* (I)** | -1.55 | -1.18 | 0 | 1.18 | 1.55 | **2.37* (I)** | -1.55 | -1.18 | -1.55 | -1.18 |
| Initiation | Verbal Fluency | Number of animals produced | 0 | 0 | -1.13 | -1.78 | -1.13 | -0.25 | 0 | 1.78 | 0.56 | 1.27 | -1.13 | 0.76 |
|  | Figural Fluency | Different items produced | **2.73* (I)** | **2.72* (I)** | 0.33 | 1.21 | 0.33 | 1.21 | 1.09 | 0.15 | -0.66 | -0.91 | -0.11 | -0.31 |
| Processing speed | Coding | Number of codes produced | 1.82 | 1.51 | 0.25 | 0.87 | 0.91 | 1.58 | 0.25 | -1.35 | -0.33 | 0.40 | -0.41 | -0.32 |
|  | Symbol Search | Number of symbols identified | 0.75 | 0.75 | 0 | 1.87 | 0.38 | 0.87 | -0.94 | -0.87 | -1.51 | 1.37 | 0.94 | -0.25 |
| Updating | Digit span | Forward span | **-2.13* (D)** | 0 | 0 | 0 | **2.13* (I)** | 0 | **2.13* (I)** | **2.75* (I)** | **-2.13* (D)** | **-2.75* (D)** | **2.13* (I)** | 0 |
|  |  | Backward span | **-2.57* (D)** | 0 | 0 | 0 | **2.57* (I)** | **1.98* (I)** | 0 | 0 | **2.13* (I)** | **1.98* (I)** | 0 | **3.95* (I)** |
|  | Letter-Number sequencing | Letter-number span | 0 | 0 | -1.14 | 0 | 1.14 | 0.70 | 1.14 | 0.70 | -1.14 | **-2.10* (D)** | 1.14 | 0.70 |
|  | Spatial Working Memory^1^ | Between Errors (8 boxes) | -1.06 | 0.71 | **-2.76* (I)** | **-3.55* (I)** | **-2.55* (I)** | **-2.84* (I)** | 0 | **-2.84* (I)** | -1.49 | -1.70 | **2.12* (D)** | -0.14 |
| Inhibition | Stroop | Inhibition ratio | **4.10* (I)** | **5.73* (I)** | -0.14 | -0.05 | -0.77 | -0.21 | 0.58 | 0.99 | 1.48 | 0.31 | -0.55 | 0.27 |
|  | Stop-Signal Task^1^ | Stop-Signal RT | **2.28*(D)** | 1.31 | 1.08 | -0.69 | -0.07 | -0.52 | -1.65 | **-2.48* (I)** | -0.23 | -0.85 | NA | -1.02 |
| Flexibility | Color Trail Test | Flexibility ratio | -1.3 | 0 | 0.93 | -0.01 | 1.27 | -0.92 | **5.52* (D)** | -0.57 | -1.4 | -1.14 | 0.2 | 1.13 |
|  | Intra-Extra Dimensional^1^ | Extra-Dimensional shift errors | 0 | NA | -0.08 | -0.10 | 1.39 | 0.90 | 0 | **-2.70* (I)** | 1.63 | -1.90 | 0.62 | 1.20 |
| Attention | CPT-3 | Omissions | 0.29 | 1.19 | 0.29 | 0 | -0.29 | -0.60 | -1.17 | **-2.98* (I)** | **2.93* (D)** | -1.79 | **-2.35* (I)** | **-4.77* (I)** |
|  |  | Commissions | -1.12 | -0.10 | 0.17 | **2.07* (D)** | 0 | -1.57 | -1.21 | -0.49 | -0.95 | -0.20 | 1.81 | 1.08 |
|  |  | HRT SD | 0.09 | -0.06 | -0.09 | 0.08 | 0.51 | 0.46 | -1.70 | **-3.79* (I)** | **2.14* (D)** | -0.78 | **-2.71*(I)** | **-5.49* (I)** |
|  |  | Perseverations | 0 | 0 | 1.30 | 1.59 | 0 | 0 | **-5.18* (I)** | **-2.38*(I)** | **-5.18*(I)** | **-3.17*(I)** | -1.30 | -0.79 |
|  |  | Detectability | -0.42 | 0.34 | **1.97* (D)** | **1.96* (D)** | -0.44 | -0.76 | **-2.29*(D)** | -1.03 | -0.23 | -0.75 | -0.19 | -0.49 |
|  |  | Variability | 0.11 | 0.13 | -0.04 | 0.04 | 0.47 | 0.42 | -1.11 | **-5.09* (I)** | 1.74 | -1.33 | -1.61 | **-8.63* (I)** |
|  |  | HRT | -0.03 | -0.39 | -0.2 | -0.19 | 0.08 | -0.54 | 0.82 | 0.92 | 0.82 | 0.55 | 0.67 | **0.77** |
|  |  | HRT Block Change | -0.11 | 0.87 | -0.35 | -0.67 | 0.49 | 0.46 | -0.93 | -0.38 | 0.15 | 1.07 | 0.81 | **0.6** |
|  |  | HRT ISI Change | 0.25 | 1.43 | 0.47 | 1.66 | 0.38 | **-2.59* (I)** | 1.66 | 0.77 | 1.36 | 1.46 | 1.08 | **1.36** |
| Learning and long-term memory | Modified 15 signs (visual modality) | Learning rate | 0 | -0.47 | -1.71 | -1.31 | 1.04 | 1.06 | 0.71 | 0.46 | -0.57 | 1.26 | 1.42 | 0.47 |
|  |  | Retention 30 min | 1.26 | 1.67 | -0.18 | -0.26 | 1.09 | 0.10 | -0.02 | -0.02 | -0.42 | -0.95 | 0.28 | -0.56 |
|  |  | Retention 1 day | 1.51 | 1.59 | -1.73 | -0.70 | 0.30 | 0.31 | -0.64 | 0.02 | **-2.27* (D)** | -0.79 | -1.01 | -0.40 |
|  |  | Retention 1 week | 1.19 | 1.89 | -0.81 | -0.13 | -0.48 | -0.45 | -1.10 | 0 | **-2.09*(D)** | -1.42 | 0.40 | 0 |
|  |  | Retention 1 month | 0.41 | 1.54 | -0.70 | -1.07 | 0.08 | 0.18 | 0.45 | 0.34 | -1.63 | -1.39 | 0 | -0.51 |
|  | Modified 15 words (verbal modality) | Learning rate | -0.57 | -0.38 | **-2.74* (D)** | **-3.06* (D)** | -0.28 | 0.38 | -0.28 | -0.13 | 1.42 | -0.57 | 0 | -0.26 |
|  |  | Retention 30 min | -0.60 | -0.25 | 1.11 | -0.08 | -1.95 | -1.73 | -0.67 | 0.31 | 0.70 | 0.58 | -0.86 | -0.50 |
|  |  | Retention 1 day | 0.60 | 0.57 | 0.57 | 0.10 | -0.78 | **-2.71* (D)** | -0.65 | -1.42 | -1.74 | -0.98 | -1.51 | -1.49 |
|  |  | Retention 1 week | 0.62 | 0.27 | NA | NA | -0.40 | -0.87 | 0 | -1.85 | -0.29 | -0.91 | 0 | -0.07 |
|  |  | Retention 1 month | **2.16* (I)** | 0.48 | NA | NA | -0.77 | **-2.00* (D)** | -0.3808 | -1.89 | 0.54 | -0.13 | -1.48 | -1.54 |

Supplementary Table S8. Magnitude of change in cognitive performance in the placebo group. Reliable improvement (I) or deterioration (D) is indicated in bold if Reliable Change Index (RCI) > ± 1.96. NA= missing data.

| **Placebo group** | | | | | | | | | | | | | |  |  |  |
| --- | --- | --- | --- | --- | --- | --- | --- | --- | --- | --- | --- | --- | --- | --- | --- | --- |
| **Cognitive domain** | **Cognitive task** | **Variable** | **Participant 1** | | **Participant 2** | | **Participant 3** | | **Participant 4** | | **Participant 5** | | **Participant 6** | | **Participant 7** | |
|  |  |  | **ST** | **LT** | **ST** | **LT** | **ST** | **LT** | **ST** | **LT** | **ST** | **LT** | **ST** | **LT** | **ST** | **LT** |
| Planning | Tower of London | Total moves | 1.20 | **2.09* (I)** | **2.40* (I)** | **2.06* (I)** | **2.40* (I)** | 0 | **2.40* (I)** | 0.69 | 1.20 | 0 | -1.20 | 0 | 1.20 | -0.69 |
| Initiation | Verbal Fluency | Number of animals produced | **2.85* (I)** | -0.78 | 1.63 | 1.56 | -0.81 | 0 | 1.63 | 0.78 | **2.04* (I)** | 1.56 | 0.41 | 0 | **3.26* (I)** | **3.52* (D)** |
|  | Figural Fluency | Different items produced | -0.19 | 1.34 | 0.78 | 0.15 | -0.19 | 0.45 | 0 | 1.19 | 0.58 | **2.24* (I)** | 1.75 | 1.94 | **-3.70* (D)** | **-2.39* (D)** |
| Processing speed | Coding | Number of codes produced | -0.86 | -0.71 | **-3.72* (D)** | 0.59 | **-2.29* (D)** | 0 | **-2.86* (D)** | -0.83 | -0.29 | 0.59 | **4.87* (I)** | **2.73* (I)** | -0.86 | -0.47 |
|  | Symbol Search | Number of symbols identified | **-2.73* (D)** | -1.37 | 0 | 0 | 0 | 0.10 | 0.73 | 0.69 | 0.36 | 1.17 | **2.19* (I)** | 1.57 | -0.18 | 0.39 |
| Updating | Digit span | Forward span | -1.76 | 0 | 0 | -1.15 | 1.76 | **2.31* (I)** | -1.76 | -1.15 | -1.76 | **2.31* (I)** | 1.76 | 0 | 0 | 0 |
|  | Digit span (inverse) | Backward span | 0.97 | 1.79 | -0.97 | -0.90 | -1.93 | -0.90 | 0 | 0.90 | -0.97 | 0.90 | -0.97 | 0 | 1.93 | 1.79 |
|  | Letter-Number sequencing | Letter-number span | -1.20 | 1.66 | -1.20 | 1.66 | -1.20 | 0 | 0 | -1.66 | 0 | 1.66 | 1.20 | 1.66 | 0 | 1.66 |
|  | Spatial Working Memory^1^ | Between Errors (8 boxes) | -1.34 | **-2.73* (I)** | 0 | 1.49 | **-2.15* (I)** | 0.25 | -0.94 | -1.73 | -0.54 | 0.99 | -0.54 | 0 | 1.34 | 0.25 |
| Inhibition | Stroop | Inhibition ratio | -1.52 | 1.23 | **-1.97* (D)** | -0.66 | **2.75* (I)** | 0.40 | -0.43 | 1.42 | -0.42 | -0.22 | 1.51 | 0.96 | **1.98* (I)** | 1.21 |
|  | Stop-Signal Task^1^ | Stop-Signal RT | 0.16 | 0.34 | -0.46 | -1.00 | -0.26 | 0.16 | 1.85 | **4.39* (D)** | -0.85 | 1.07 | -1.56 | NA | 1.19 | 0.95 |
| Flexibility | Color Trail Test | Flexibility ratio | 1.34 | 0.35 | -1.39 | -1.08 | 0.74 | 1.24 | -0.59 | 0.90 | 1.71 | -1.45 | -0.34 | 0.12 | 0.21 | 0.67 |
|  | Intra-Extra Dimensional^1^ | Extra-Dimensional shift errors | -0.90 | -0.71 | 0.09 | 0.07 | -1.16 | -1.38 | -1.68 | -1.57 | 1.42 | 1.12 | 0 | 0 | -0.09 | 0.22 |
| Attention | CPT-3 | Omissions | 0.81 | 0 | 0.27 | 0.60 | 0 | -0.60 | 0 | 0 | -1.35 | **-3.00* (I)** | -1.89 | **-2.40* (I)** | 0 | **2.40* (D)** |
|  |  | Commissions | 0.19 | -0.29 | 1.72 | **2.00* (D)** | -1.05 | -0.48 | 1.72 | **2.00* (D)** | 0.19 | -0.19 | 0.67 | -1.33 | 0.67 | 0.95 |
|  |  | HRT SD | 0.13 | -0.02 | -0.07 | 0.12 | -0.71 | -0.90 | -0.14 | -0.25 | **-3.16* (I)** | **-4.43* (I)** | **-2.58* (I)** | **-2.80* (I)** | 1.25 | 0.80 |
|  |  | Perseverations | 0 | 0 | 0 | 0 | 0 | 0 | -1.62 | -0.82 | 0 | 0 | -1.62 | -0.82 | **4.87* (D)** | **7.37* (D)** |
|  |  | Detectability | 1.01 | -0.54 | 1.58 | **2.26* (D)** | -1.27 | -1.20 | 0.50 | 0.43 | -1.23 | -1.39 | -1.21 | -1.57 | **2.33* (D)** | **2.32* (D)** |
|  |  | Variability | 0.04 | 0 | -0.04 | -0.02 | -0.50 | -1.06 | -0.06 | -0.18 | **-1.80* (I)** | **-3.73* (I)** | -1.57 | **-2.47* (I)** | 1.41 | 0.41 |
|  |  | HRT | -0.45 | -0.17 | -0.52 | 0.99 | 0 | 0.99 | -0.69 | -0.22 | -0.63 | -1.57 | 0.51 | -0.98 | 0.29 | 0.15 |
|  |  | HRT Block Change | 0.46 | 0 | -0.69 | 1.57 | 0.52 | 1.57 | -0.36 | -1.19 | -0.06 | -0.65 | -1.18 | -1.24 | 0.23 | 0.15 |
|  |  | HRT ISI Change | **-2.74*(I)** | **-2.48*(I)** | -1.22 | **3.50* (D)** | -1.52 | -0.57 | -1.70 | -1.13 | -2.00 | 0.18 | 4.39 | 1.18 | -1.93 | 1.70 |
| Learning and long-term memory | Modified 15 signs (visual modality) | Learning rate | 0.35 | -0.43 | NA | NA | **-2.30* (D)** | -1.30 | 1.06 | -0.65 | 0.53 | -1.43 | -0.35 | 0.43 | 1.42 | 1.30 |
|  |  | Retention 30 min | -1.31 | -1.41 | NA | NA | 0.97 | 0.27 | -0.49 | -1.88 | -0.14 | 1.61 | 1.48 | 0.31 | 0.93 | 1.80 |
|  |  | Retention 1 day | -1.78 | -1.05 | NA | NA | **-5.40* (D)** | -0.19 | **-3.56* (D)** | -0.96 | **-2.04* (D)** | 0.44 | -0.45 | 0.05 | **-2.28* (D)** | -0.77 |
|  |  | Retention 1 week | 0.12 | -1.16 | NA | NA | 0.28 | -0.47 | -0.93 | -0.53 | NA | NA | -0.22 | -0.85 | **-2.81* (D)** | **-2.33* (D)** |
|  |  | Retention 1 month | -0.37 | -0.36 | NA | NA | 1.78 | -0.48 | **-2.69* (D)** | 0.52 | NA | NA | -0.23 | -0.23 | 0.38 | -1.69 |
|  | Modified 15 words (visual modality) | Learning rate | 0.21 | 0.35 | 1.24 | NA | 1.06 | -0.59 | **-2.49* (D)** | **-3.15* (D)** | -1.24 | 1.05 | -0.41 | -0.35 | -1.66 | **-2.03* (D)** |
|  |  | Retention 30 min | -0.5 | -1.00 | -0.38 | NA | 0.06 | 0.76 | 0 | 0.83 | -0.75 | 0 | -1.10 | **-2.44* (D)** | **2.14* (D)** | 1.41 |
|  |  | Retention 1 day | 0.47 | 0.25 | 1.68 | NA | -0.97 | -0.57 | -1.12 | **-2.09* (D)** | 0.28 | 0.42 | -1.01 | 0.58 | 0.50 | -0.10 |
|  |  | Retention 1 week | -0.73 | **-2.10* (D)** | 0.95 | NA | 0.65 | -0.46 | 0.38 | 0 | -0.76 | **-4.45* (D)** | -0.65 | **-2.15* (D)** | -1.14 | **-3.82* (D)** |
|  |  | Retention 1 month | **-2.95* (D)** | -1.15 | -1.4 | NA | -1.04 | -0.66 | 0.47 | 0.38 | **-2.80* (D)** | **-3.06* (D)** | **-2.51* (D)** | -1.29 | **-3.70* (D)** | **-2.65* (D)** |

-
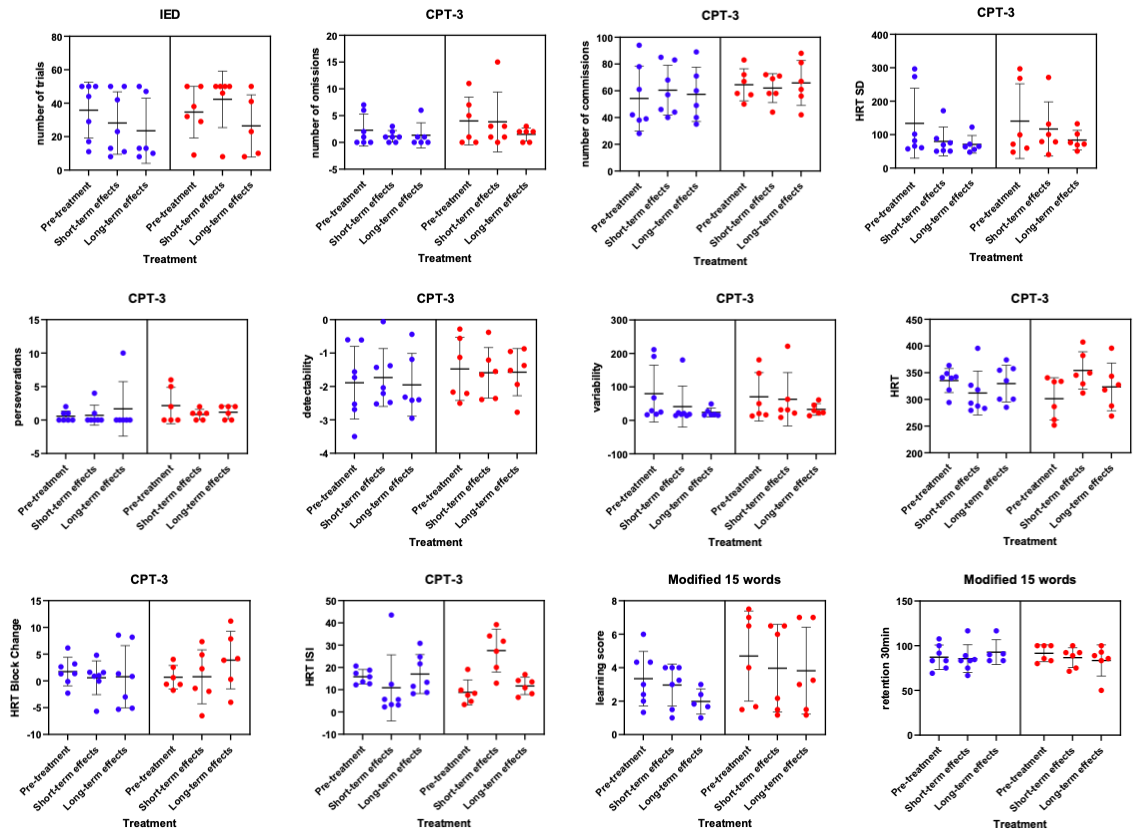

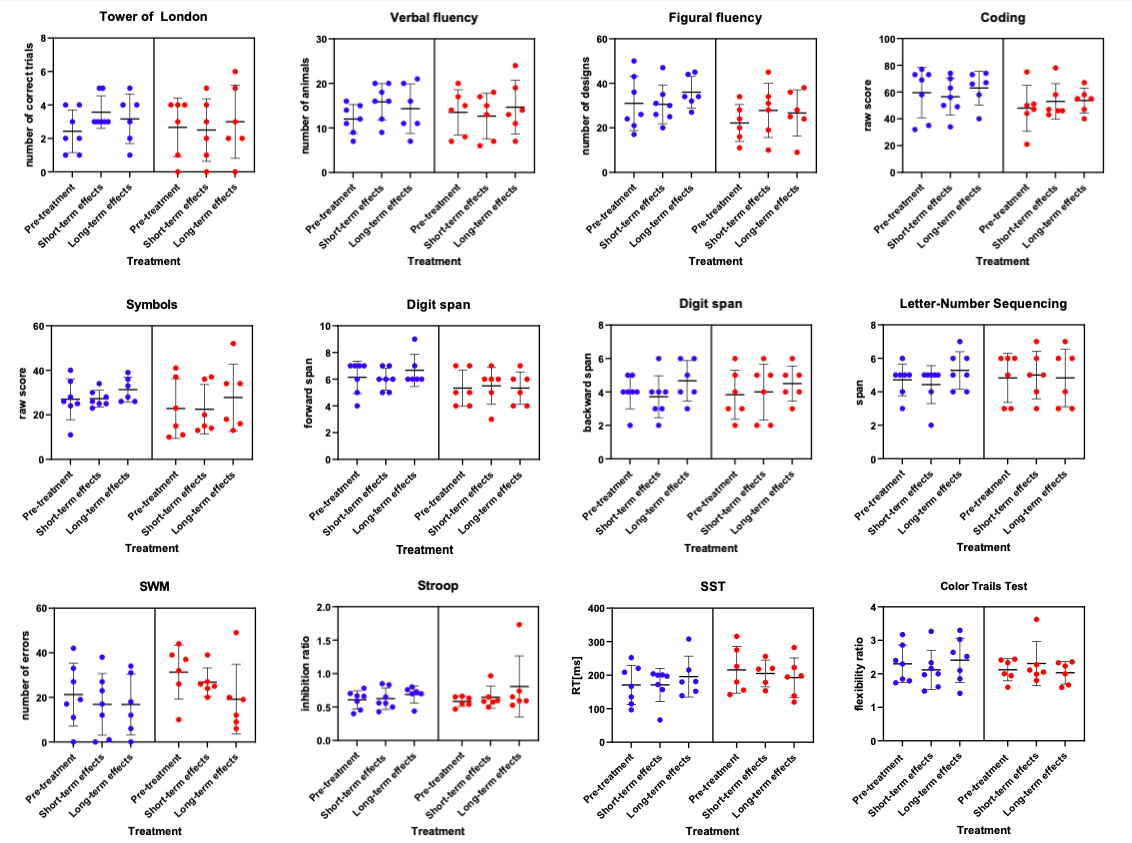
 Supplementary Figures (S1-S2)


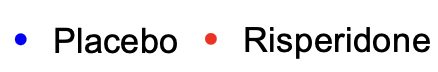

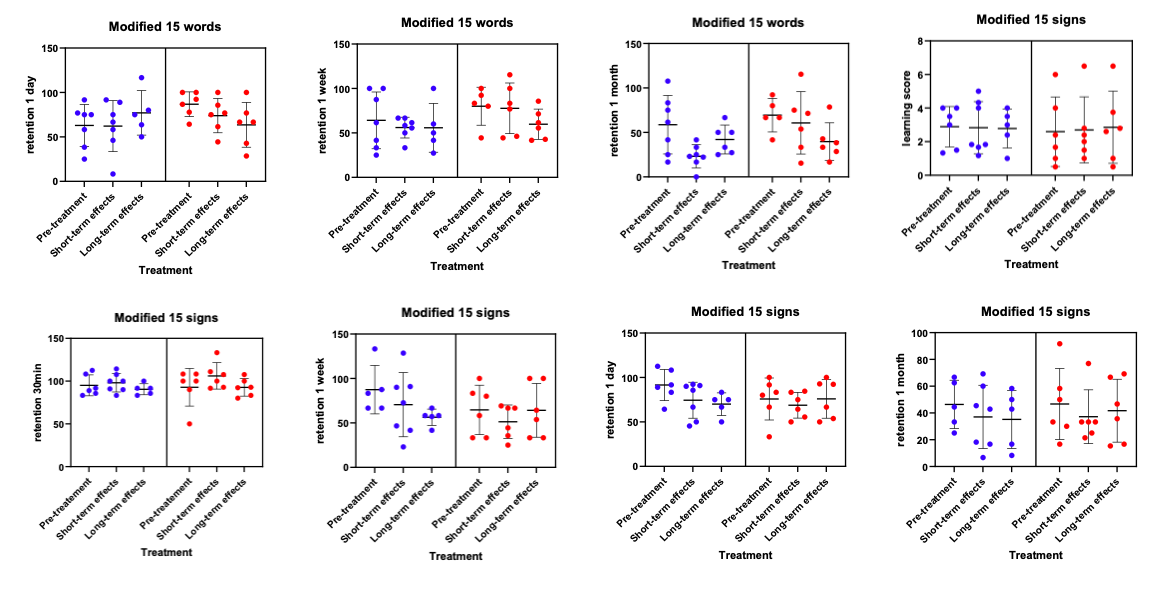


Supplementary Figure S1. Distribution of scores in cognitive variables in both the placebo (blue) and the risperidone (red) groups. Detailed information regarding each cognitive measure is described in Supplementary Table S2.


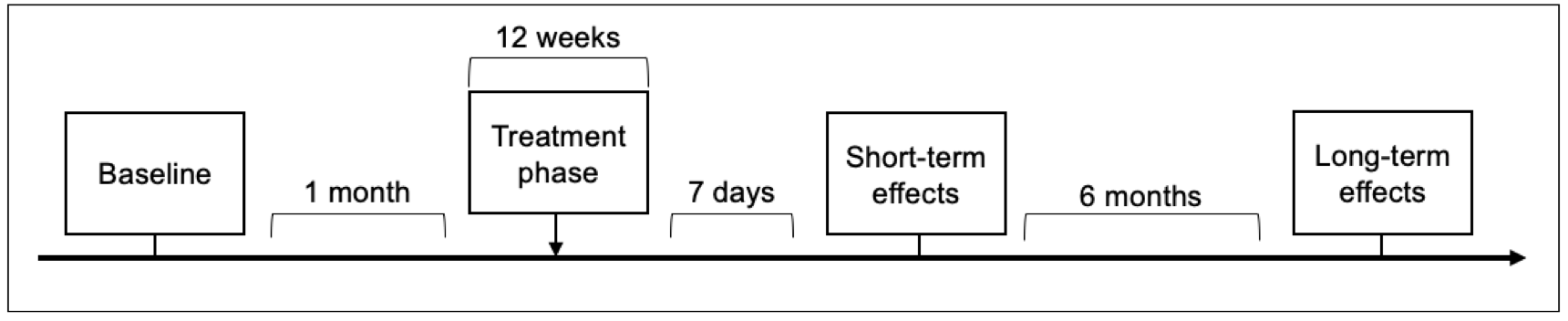


Supplementary Figure S2. Study design
